# Inhibitory effect of a novel peptide, H-RN, on keratitis induced by LPS or poly(I:C) in vitro and in vivo via suppressing NF-κB and MAPK activation

**DOI:** 10.1186/s12967-017-1121-z

**Published:** 2017-01-26

**Authors:** Shaopin Zhu, Xun Xu, Lili Wang, Li Su, Qing Gu, Fang Wei, Kun Liu

**Affiliations:** 1 Department of Ophthalmology, School of Medicine, Shanghai First People’s Hospital, Shanghai Jiao Tong University, No. 100, Haining Road, Shanghai, People’s Republic of China; 2 Shanghai Key Laboratory of Fundus Disease, No. 100, Haining Road, Shanghai, People’s Republic of China

**Keywords:** Keratitis, Corneal inflammation, Peptide, LPS, Poly(I:C)

## Abstract

**Background:**

Keratitis is a common cause of blindness. Current anti-inflammatory drugs used in keratitis have profound side effects. Small peptides derived from endogenous proteins potentially display both desired efficiency and safety. We identified an 11-amino-acid peptide, H-RN, from hepatocyte growth factor (HGF), an endogenous protein with anti-inflammatory properties. We evaluated the effects of H-RN in keratitis in vitro and in vivo.

**Methods:**

In vitro, corneal fibroblasts were stimulated with LPS or poly(I:C), surrogates for bacteria and viruses. Inflammatory cytokines, intercellular cell adhesion molecule-1 (ICAM-1), translocation of NF-κB p65, activation of IκBα, NF-κB, and MAPKs were detected. In vivo, keratitis in rats was induced by LPS. Clinical, histological observation, and quantification of cytokines in the cornea were conducted. H-RN safety was measured by cell viability, clinical, histological, and microstructural observations.

**Results:**

H-RN inhibited IL-6, monocyte chemotactic protein-1(MCP-1), Interferon- γ(IFN-γ), and ICAM-1 expression triggered by LPS or poly(I:C), alleviated the clinical manifestation and reduced the clinical score in keratitis in vivo. The histological disorder and proinflammatory cytokines of the cornea were also reduced. The translocation of NF-κB and phosphorylation of IκBα, NF-κB, p38, JNK, and ERK were significantly inhibited by H-RN. No sign of toxicity was observed.

**Conclusions:**

H-RN effectively attenuated keratitis in vivo and in vitro induced by LPS or poly(I:C) through blocking NF-κB and MAPK signaling pathways. It may be a promising and safe agent in treating keratitis.

## Background

Inflammatory corneal diseases could cause severe damage to the physical structure and visual functions, leading to opacity, scarring, perforation of the cornea, and even permanent vision loss [1]. Bacteria and viruses are the most common infectious agents that affect the ocular surface and result in inflammatory damages. Once exposed to those insults, the immune system of the cornea is triggered, followed by the release of cytokines and infiltration of inflammatory cells [2, 3]. Even though antibiotics and antivirals eliminate many of the microbes, the inflammatory process initiated by the infection won’t stop immediately, causing lasting and recurrent destructive actions [4]. Anti-inflammatory or immunosuppressive agents are needed to limit the immune response. However, such drug options are relatively limited and generate profound side effects, including glaucoma, cataract, and delay of healing [5]. Identifying and developing new drugs which display both desired efficiency and satisfactory safety is a challenge. Due to the specific structure of the eye, small peptides with lower immunogenicity, higher penetration capability, and better cost-effectiveness are preferred and display certain merits [5–7].

Hepatocyte growth factor is a multifunctional protein that was first found as a potent mitogenic protein for mature hepatocytes and later demonstrated its role in cell protection, regeneration, tissue survival, and repair. With emerging evidence that it protects against key events in autoimmune and inflammatory disorders, approaches have been made to explore the possibility of its therapeutic use against diseases such as autoimmune neuroinflammation, rheumatoid arthritis, and asthma [8–10]. However, as for ocular diseases, such agents of large molecules can hardly penetrate the ocular tissues and barriers to taking effects. In this regard, small peptides derived from key active parts of certain functional proteins display promising potential to target ocular disorders [6, 11, 12].

Previously, we have identified an 11-amino-acid peptide, H-RN (RNPRGEEGGPW), from the Kringle 1 domain of HGF using biological information technology and have studied its inhibitory effect in uveitis and neovascularization [13, 14]. In fact, among ocular surface diseases, keratitis has a much higher incidence rate than uveitis. Besides, the cells, pathogenic factors, and mechanisms involved in the two diseases are not the same. Whether H-RN exhibits a protective effect on corneal inflammation was not explored yet. Besides, previously, only LPS was used in the experiments, and it was still unknown whether H-RN had an effect on poly(I:C)-induced inflammation, which was important in studying virus-related inflammation. To investigate H-RN’s effect on keratitis, the corneal fibroblasts, the primary resident cells of the cornea and the most vulnerable cells in keratitis, were studied. Not only LPS, a surrogate for bacteria but also poly(I:C), a synthetic analog of viral double-stranded RNA, were applied for the first time in our research to see whether H-RN interferes with the virus- or bacteria-related inflammation. Also, an animal model of LPS-induced corneal inflammation was conducted and administrated with H-RN to study its effect in vivo. Moreover, the possible mechanisms concerning inflammation signaling were also investigated.

## Methods

### Peptide synthesis

Peptide H-RN (RNPRGEEGGPW) and a scrambled control peptide H-GP (GPERWRGPNGE) were synthesized by China Peptides Co., Ltd (Shanghai, China) using the high-efficiency solid-phase method. The final product reached purity over 98% that was characterized by analytical high-performance liquid chromatography and mass spectrometry. All the peptides were kept in −30 °C and dissolved in the medium before the experiments.

### Cell culture and induction of inflammation in corneal fibroblasts—in vitro model

Human corneal fibroblasts (Catalog #6520) were obtained from ScienCell company (ScienCell, Carlsbad, CA, USA). Cells were cultured in Fibroblast Medium containing 2% FBS, 1% fibroblast growth supplement (FGS), and 1% penicillin/streptomycin solution as recommended by ScienCell. Corneal fibroblasts were cultured at 37 °C in 5% humidified CO2. Cells between passages 4–8 were used in the experiments. LPS from *Escherichia coli* (Sigma-Aldrich, Inc., St. Louis, MO, USA) (10 μg ml^−1^) and poly(I:C) (Invivogen, San Diego, California, USA) (10 μg ml^−1^) were used to induce an inflammatory reaction in corneal fibroblasts. Different concentrations of H-RN (10 μM, 50 μM, 100 μM), H-GP (100 μM), and dexamethasone(DEX) (100 μM) (Shanghai General Motors Pharmaceutical Industry Company Limited, China) were added to different groups as interventions.

### Induction of keratitis in rats—in vivo model

All experiments were consistent with the ARVO Statement for the Use of Animals in Ophthalmic and Vision Research. Male Wistar rats (200–240 g) were procured from Shanghai Laboratory Animal Center, Chinese Academy of Sciences and were maintained in a 12 h light/12 h dark cycle. Food and water were supplied ad libitum. The rats were divided into appropriate experimental groups by complete randomization. Keratitis was induced by injecting LPS into the corneal stroma of the rats as described before [2, 15]. Briefly, after anesthetization of the rats, a tunnel to the central stroma of the cornea was created by a 33-gauge needle (Hamilton Co., Reno, NV, USA). Then another 33-gauge needle attached to a 5-μl syringe was used to inject 10 μg LPS (diluted in 5 μl saline) into the stroma through the tunnel. Rats in the control group only received a 5-μl saline stromal injection. LPS group was subconjunctivally injected with 20 μl saline, while peptide groups received a subconjunctival injection of 20 μl saline containing 10, 20 or 50 μg H-RN, or 50 μg H-GP separately. Besides, we also administered eye drops (20 μl) containing 10, 20, or 50 μg peptides to the LPS-induced rats every 4 h for 24 h. DEX was used as the drug control.

### ELISA assay

IL-6, MCP-1, and IFN-γ of corneal fibroblasts (n = 7) and rat corneas (n = 7P) were measured by ELISA assay. Cell supernatants of different groups were collected, centrifuged, and stored at −80 °C. The rat corneas were isolated carefully and homogenized in 100-μl sterile PBS, which were then centrifuged at 15,000 rpm for 20 min at 4 °C. Finally, the supernatants were collected and stored at −80 °C. Both samples from cells and corneas were quantified by different ELISA Kits (R&D Systems, Minneapolis, MN, USA) according to the manufacturer’s directions. All measurements were performed in triplicate.

### RT-PCR analysis

RT-PCR was performed to determine the mRNA level of IL-6, MCP-1, IFN-γ, and ICAM-1, both in the cells (n = 7) and the corneas (n = 7). The total RNA of corneal fibroblasts and rat cornea were extracted using Trizol reagent (Invitrogen, Carlsbad, CA, USA) and detected by performing RT-PCR on in a ViiA 7 Detection System (Applied Biosystems, Foster City, CA, USA). The amount of IL-6, MCP-1, IFN-γ, or ICAM-1 mRNA was normalized by that of GAPDH mRNA and is presented in arbitrary units (1 unit corresponds to the value for cells in control group).

The primer used were as follows:

human IL-6:(forward) 5′- TTCGGTCCAGTTGCCTTCT-3′ and (reverse) 5′-GGTGAGTGGCTGTCTGTGTG-3′; human MCP-1: (forward) 5′-ATCAATGCCCCAGTCACCT-3′ and (reverse) 5′-TCCTGAACCCACTTCTGCTT-3′; human IFN-γ: (forward) 5′-GAGTGTGGAGACCATCAAGGA-3′ and (reverse) 5′-GTATTGCTTTGCGTTGGACA-3′; human ICAM-1 (forward) 5′-TCACCTATGGCAACGACTCC-3′ and (reverse) 5′-CAGTGTCTCCTGGCTCTGGT-3′; rat IL-6: (forward) 5′-AGTTGCCTTCTTGGGACTGA-3′ and (reverse) 5′-ACTGGTCTGTTGTGGGTGGT-3′; rat MCP-1(forward) 5′-TCACCTGCTGCTACTCATTCA-3′ and (reverse) 5′-CCATTCCTTATTGGGGTCAG-3′.

### Immunofluorescence detection

For detection of the p65 subunit of NF-κB and the expression of ICAM-1 in the cells, immunofluorescence was performed (n = 5). Corneal fibroblasts were seeded on coverslips cultured in fibroblast medium containing 2% FBS and were changed to serum-free medium overnight when the cells reached 80–90% confluence. Then the cultured corneal fibroblasts were stimulated with LPS (10 μg ml^−1^) or poly(I:C) (10 μg ml^−1^) for 1 h to detect NF-κB p65 and 24 h to detect ICAM-1. At the same time, cells were incubated with or without H-RN (10, 50, 100 μM), or H-GP (100 μM). After being washed, the cells were fixed with methanol: acetone (1:1), permeabilized by 0.1% Triton X-100 for 10 min, and blocked with PBS containing 2% bovine serum albumin for 30 min. Then the cells were incubated with primary antibody against NF-κB p65 (rabbit monoclonal antibodies to NF-κB p65, 1:1000, Cell Signaling Technology, Beverly, MA, USA) and ICAM-1 (mouse monoclonal antibodies to ICAM-1, 1:500, R&D, Minneapolis, MN, USA) overnight at 4 °C. The secondary antibodies (Alexa Fluor 555, 1:500, Molecular Probes, Eugene, OR, USA,) against rabbit or mouse IgG were incubated with the cells for 1 h. The cells were stained with DAPI before being viewed and captured with a confocal laser scanning microscope (Zeiss LSM510; Carl Zeiss, Thornwood, NY, USA).

### Western blot analysis

Cells (n = 5) were pretreated with different concentrations of H-RN for 30 min and then incubated with LPS or poly(I:C) (both 10 μg ml^−1^) for 1.5 h (for NF-κB and MAPKs) or 24 h (for ICAM-1). After that, cells were washed in ice-cold PBS, collected, lysed, and centrifuged. Protein concentrations were determined by a Bradford protein assay (Bio-Rad, Munich, Germany). The lysates of the samples were loaded and separated by SDS-PAGE and then electrophoretically transferred to polyvinylidene difluoride membranes. The membranes were blocked with 5% BSA and incubated with primary antibodies overnight at 4 °C, which included ICAM-1 (R&D, Minneapolis, MN, USA), NF-κB p65, p-NF-κB p65, IκBα, p-IκBα, ERK, p-ERK, JNK, p-JNK, p38, and p-p38 (Cell Signaling Technology, Beverly, MA, USA). After washing, the membranes were further detected with secondary anti-rabbit or anti-mouse antibodies (Santa Cruz, CA, USA). Images of the bands were obtained using Amersham Imager 600 system (GE Healthcare Bio-Sciences AB, Uppsala, Sweden). Analyses of the relative protein levels were evaluated by Image J software (NIH). The experiments were repeated at least three times to ensure reproducibility.

### Clinical evaluation

Examination with a biomicroscope on a masked basis was performed on all the rats 24 h after LPS stimulation. The severity of the stromal keratitis was graded in a blinded fashion with a scoring system described previously [16]: 0.0 = Normal; 0.5–0.9 = Mild edema, not diffuse, no haze; 1.0–1.9 = Significant edema, slight haze, iris clearly visible; 2.0–2.9 = Gross edema, stromal swelling, cloudy, diffuse, can see anterior chamber, iris visible; 3.0 = Severe stromal edema, very cloudy, cannot see anterior chamber, pupillary border no longer distinct; 4.0 = Opaque cornea, anterior chamber structure not visible.

### Histopathological examination

Corneas were histologically studied to evaluate the effect of H-RN on stromal keratitis. Eyes were removed and fixed in 4% paraformaldehyde overnight at room temperature. Sagittal serial sections were taken and stained using hematoxylin and eosin (H&E). The number of infiltrated cells in the cornea per field was evaluated under light microscopy in a masked fashion and manually counted by two independent researchers of four random fields.

### Safety assessment

To evaluate the irritation and ocular safety of H-RN solutions, both cell examination and in vivo assessment were developed. 3-(4,5-dimethylthiazol-2-yl)-5-(3-carboxymethoxypehenol)-2-(4-sul-phophenyl)-2H-tetrazolium (MTS) method was used to determine the viability of cells. After incubation with different concentration of H-RN (10, 50, 100, 200 μM) for 24 h, corneal fibroblasts were treated with 20 ml of the MTS reagent (Promega Corporation, Madison, USA) for 3 h at 37 °C and then a microtiter plate reader (Bio-Rad, Model 680, Hercules, CA, USA) was used to measure the optical density. Moreover, the possible toxicity was also accessed everyday by biomicroscope. One week after rats received a subconjunctival injection of 20 μl saline containing 100 μg H-RN, histopathological examination, and transmission electron microscope tests were performed.

### Statistical analysis

Results are expressed as mean ± SD and were evaluated by analysis of variance (ANOVA) followed by Bonferroni’s post hoc test using the SPSS 16.0 software. In cases of nonparametric data distribution, the Kruskal–Wallis test was performed followed by Mann–Whitney U test. A *p* value less than 0.05 was considered statistically significant.

## Materials

Peptide H-RN (RNPRGEEGGPW) and a scrambled control peptide H-GP (GPERWRGPNGE) were synthesized by China Peptides Co., Ltd (Shanghai, China). Human corneal fibroblasts (Catalog #6520) and Fibroblast Medium were obtained from ScienCell company (Carlsbad, CA, USA). LPS from Escherichia coli was bought from Sigma-Aldrich Inc (St. Louis, MO, USA), poly(I:C) was from Invivogen (San Diego, California, USA) and dexamethasone (DEX) was from Shanghai General Motors Pharmaceutical Industry Company Limited (Shanghai, China). ELISA Kits and primary antibody against ICAM-1 were from R&D (Minneapolis, MN, USA). Trizol reagent was from Invitrogen (Carlsbad, CA, USA). RT-PCR was performed with a ViiA 7 Detection System (Applied Biosystems, Foster City, CA, USA). Primary antibodies against NF-κB p65, p-NF-κB p65, IκBα, p-IκBα, ERK, p-ERK, JNK, p-JNK, p38, and p-p38 were obtained from Cell Signaling Technology (Beverly, MA, USA). Alexa Fluor 555 secondary antibodies were bought from Molecular Probes (Eugene, OR, USA,). Secondary anti-rabbit or anti-mouse antibodies used in western blot were from Santa Cruz (CA, USA). Immunofluorescence detection was performed with a confocal laser scanning microscope (Zeiss LSM510; Carl Zeiss, Thornwood, NY, USA). Images of the bands were obtained using Amersham Imager 600 system (GE Healthcare Bio-Sciences AB, Uppsala, Sweden). Analyses of the relative protein levels were evaluated by Image J software (NIH). MTS reagent was obtained from Promega Corporation (Madison, USA) and MTS assay was determined with a microtiter plate reader (Bio-Rad, Model 680, Hercules, CA, USA).

## Results

### Inhibition of IL-6, MCP-1, and IFN-γ from corneal fibroblasts induced by LPS or poly(I:C)

**Fig. 1:**
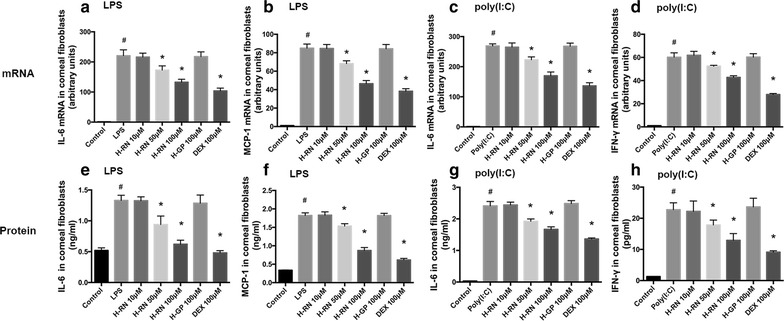
Effect of H-RN on the production of IL-6, MCP-1 and IFN-γ in corneal fibroblasts. Cells were stimulated by LPS (10 μg ml^−1^) or poly(I:C) (10 μg ml^−1^) and incubated with H-RN (10, 50, 100 μM), H-GP (100 μM), and DEX (100 μM) for 6 h (for RT-PCR) or 24 h (for ELISA). The mRNA (**a**–**d**) and protein (**e**–**h**) level of IL-6, MCP-1, and IFN-γ were analyzed by RT-PCR and ELISA. Data are expressed as mean ± SD. ^#^p < 0.05 compared with control group; *p < 0.05 compared with LPS or poly(I:C) group; DEX, dexamethasone

Corneal fibroblasts exposed to LPS or poly(I:C) were treated with or without H-RN. The inflammatory cytokines including IL-6, MCP-1, and IFN-γ were markedly released from corneal fibroblasts. We examined the mRNA and protein level of the above cytokines using RT- PCR and ELISA methods. As is shown in Fig. 1, LPS induced IL-6 and MCP-1 increases, which was significantly reduced by treatment with H-RN and DEX not only at the mRNA (Fig. 1a–d) but also the protein level (Fig. 1e–h). Poly(I:C) induced IL-6 and IFN-γ increases and was also remarkably inhibited by H-RN and DEX. H-GP had no effect on corneal fibroblasts in the above experiments.

### Suppression of the mRNA and surface expression of ICAM-1 in corneal fibroblasts

**Fig. 2:**
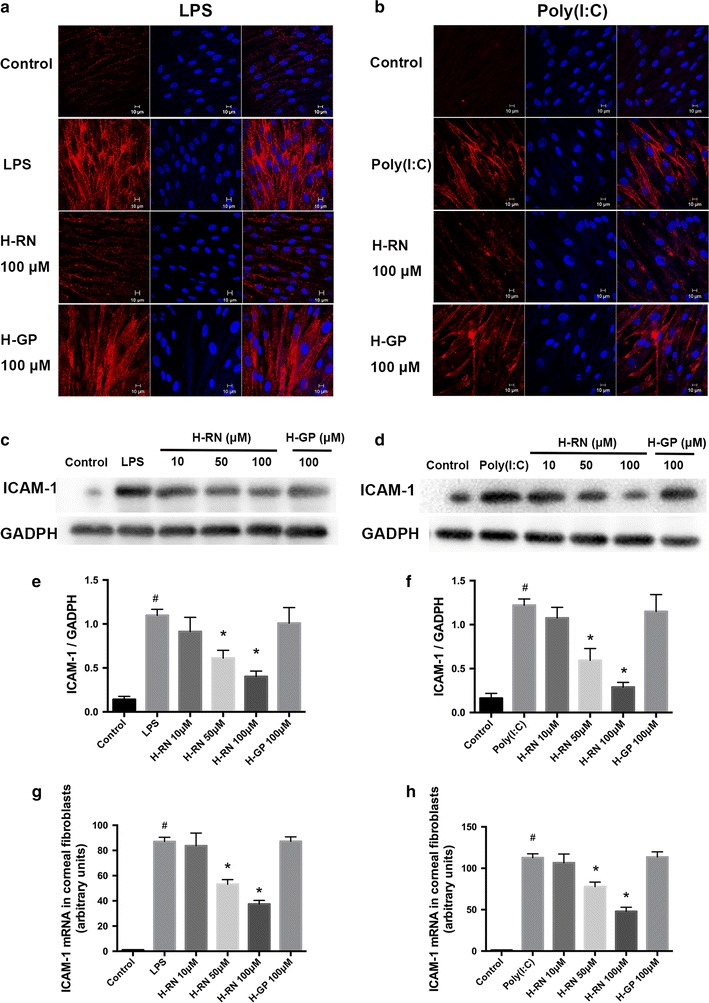
Suppression by H-RN of mRNA and surface expression of ICAM-1 in corneal fibroblasts. Cells were stimulated by LPS (10 μg ml^−1^) or poly(I:C) (10 μg ml^−1^) and incubated with or without peptides. The expression of ICAM-1 was measured by immunofluorescence (**a**, **b**), *Western blot* (**c**–**f**), and RT-PCR (**g**, **h**). Data are expressed as mean ± SD. ^#^p < 0.05 compared with control group; * p < 0.05 compared with LPS or poly(I:C) group. Nucleus (DAPI, *blue*), ICAM (in *red*)

We performed RT- PCR, immunofluorescence, and Western blot to determine the effect of H-RN on the ICAM-1, one of the key adhesion molecules involved in inflammation. As is shown in Fig. 2, the fluorescence intensity (Fig. 2a, b), the band abundance (Fig. 2c–f), and the mRNA relative level (Fig. 2g, h) of ICAM-1 were obviously elevated after stimulation with LPS or Poly(I:C). Incubation with H-RN resulted in the significant suppression of ICAM-1 in a dose-dependent manner.

### Attenuation of clinical manifestation and reduction of clinical score

**Fig. 3:**
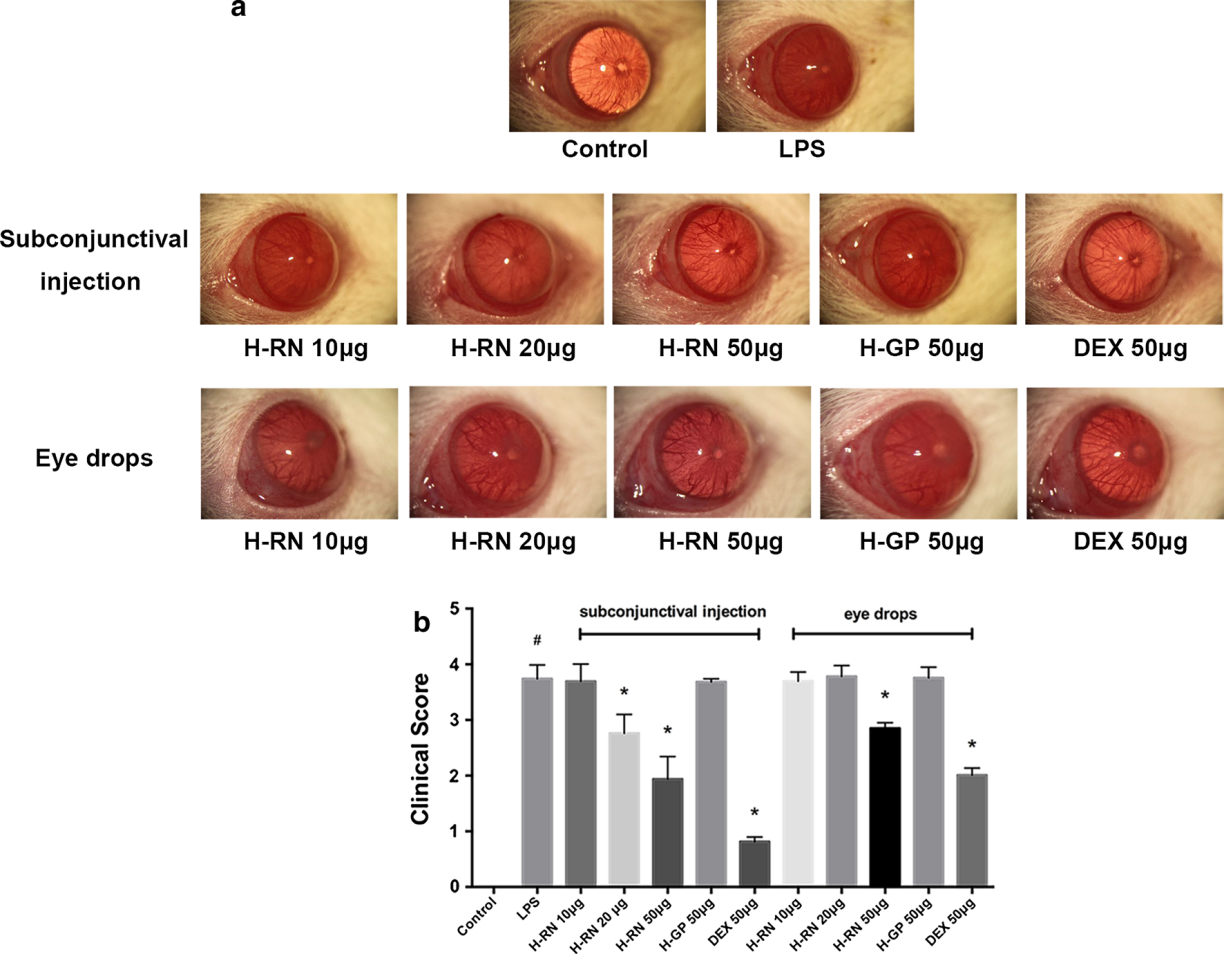
Attenuation of clinical manifestation (**a**) and clinical score (**b**) by H-RN in LPS-induced keratitis in rats. Rats except those in control group (PBS injection) were injected with LPS (10 μg diluted in 5-μl saline) into the corneal stroma and received subconjunctival injection or eye drops of H-RN or H-GP, DEX. Eyes were examined and scored 24 h later. Data are expressed as mean ± SD. ^#^p < 0.05 compared with control group; *p < 0.05 compared with LPS group; DEX, dexamethasone

After injection of LPS for 24 h, a distinctive inflammatory response was observed in rat corneas. In the LPS group, rats showed severe corneal edema, opacity, and swelling with a significantly increased clinical score compared to normal rats (Fig. 3). In the H-RN treated group, all those symptoms were relieved greatly, especially in the subconjunctival injection groups. The clinical score was markedly reduced by subconjunctival injection or eye drops of H-RN. In the subconjunctival injection group, the inhibition effect of H-RN was more significant, and the clinical score was lower than the topical administration group. In contrast, the scrambled peptide, PAPsc, had no effect on corneal inflammation.

### Suppression of histopathological disorders of the corneal tissue

**Fig. 4:**
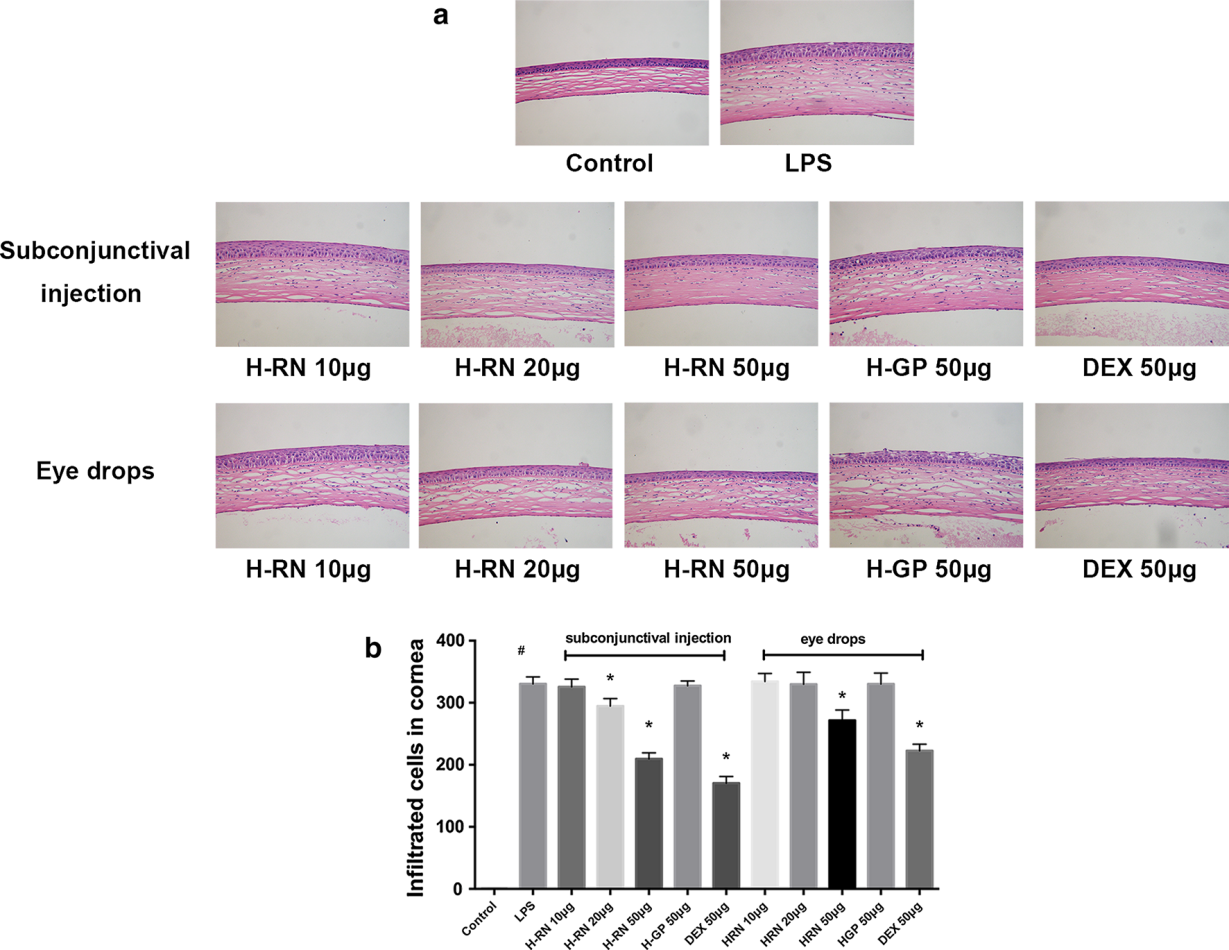
Effect of H-RN on histological disorders and cell infiltration in the cornea. Eyeballs were collected 24 h after LPS injection. **a** Histological observation **b** number of infiltrated inflammatory cells. Original magnification (A–J) ×100 Data are expressed as mean ± SD. ^#^p < 0.05 compared with control group; *p < 0.05 compared with LPS group; *DEX* dexamethasone

Furthermore, we observed the histological manifestations between different groups. As shown in Fig. 4, LPS disrupted the structure of the normal cornea, leaving the tissue obviously thickened, swollen, and the cells arranged in disorder with an infiltration of large amounts of inflammatory cells. The pathological results were consistent with clinical features. In H-RN- and DEX-treated rats, especially those who received a subconjunctival injection, the disorders of the corneal tissue were significantly ameliorated with fewer infiltrated cells. There is no difference between the PAPsc and LPS groups.

### Reduction of mRNA and protein expression of inflammatory cytokines in rat corneas

**Fig. 5:**
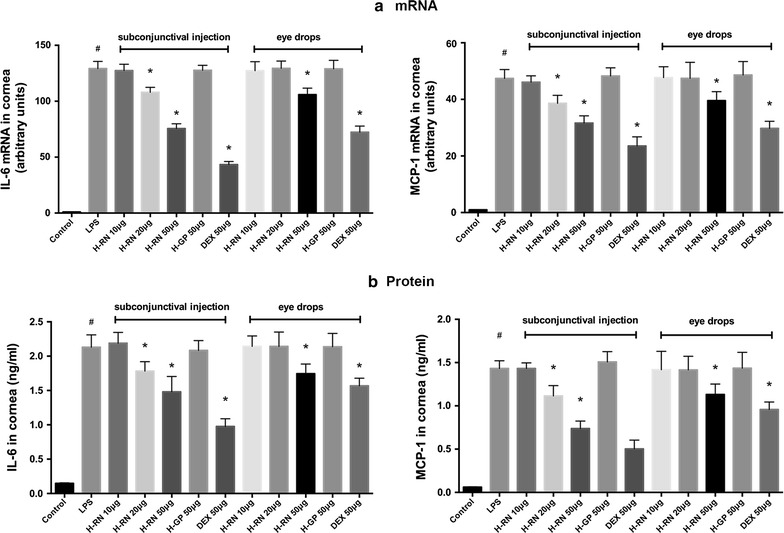
Effect of H-RN on mRNA and protein level of inflammatory cytokines in rat corneas. The mRNA and protein level of IL-6 and MCP-1 of rat corneas were analyzed by RT-PCR (**a**) and ELISA (**b**). Data are expressed as mean ± SD. ^#^p < 0.05 compared with control group; *p < 0.05 compared with LPS group; *DEX* dexamethasone

We also conducted RT-PCR and ELISA to identify the level of IL-6 and MCP-1 in rat corneas. By the results from corneal fibroblasts, both the mRNA transcription (Fig. 5a) and protein expression (Fig. 5b) of the two cytokines were significantly inhibited by H-RN compared with the control group.

### Effects of the LPS- or poly(I:C)-induced phosphorylation of IκBα, p65, and p65 translocation in the corneal fibroblasts

**Fig. 6:**
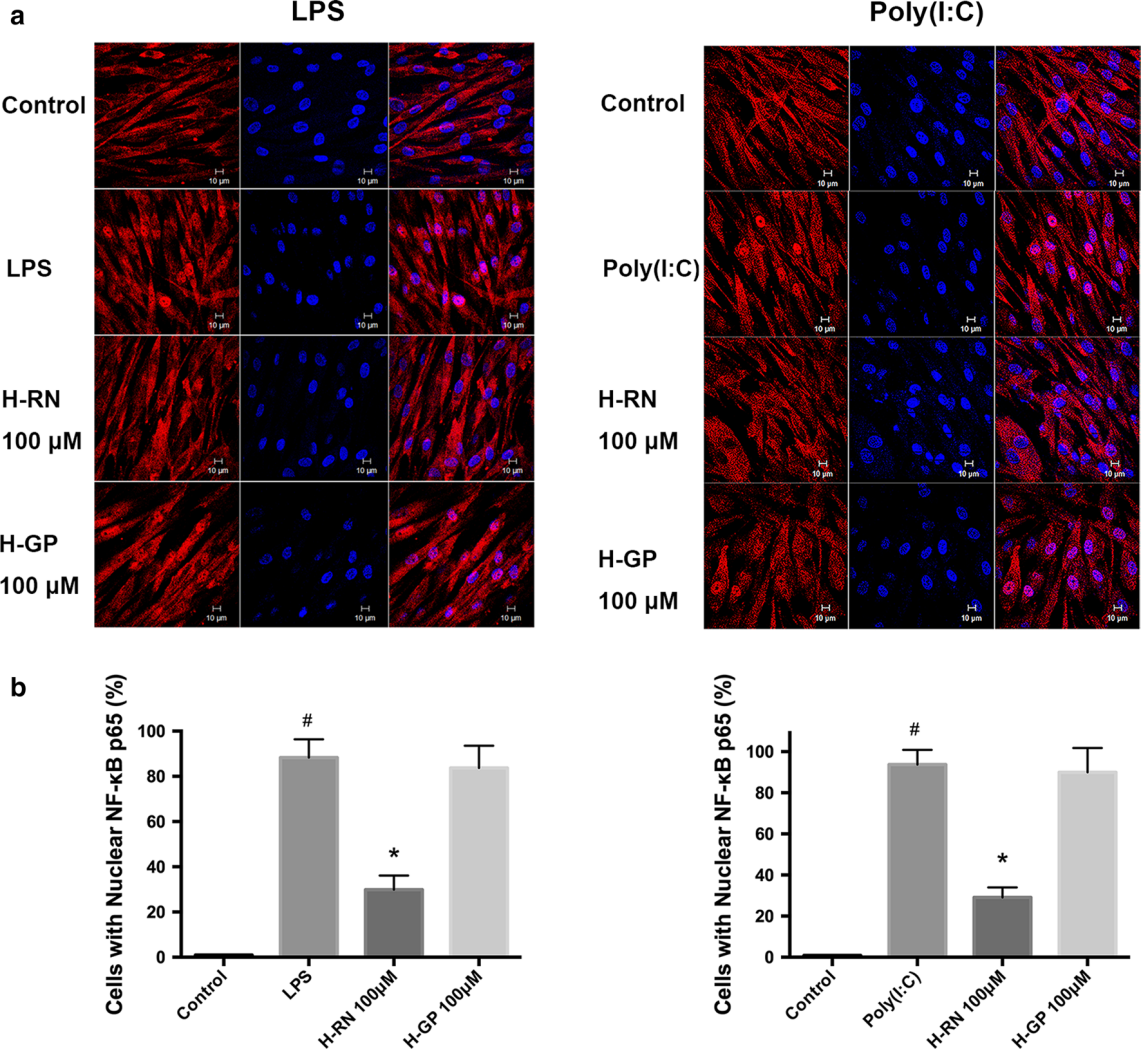
Reduction of translocation of p65 subunit of NF-κB by H-RN in corneal fibroblasts induced by LPS or poly(I:C). Translocation of p65 subunit of NF-κB was observed by immunofluorescence (**a**). The percentage of the number of translocated cells (**b**) was calculated by counting the number of cells with nuclear translocation in a masked fashion. Data are expressed as mean ± SD. ^#^p < 0.05 compared with control group; *p < 0.05 compared with LPS or poly(I:C) group. Nucleus (DAPI, *blue*), ICAM (in *red*)

**Fig. 7:**
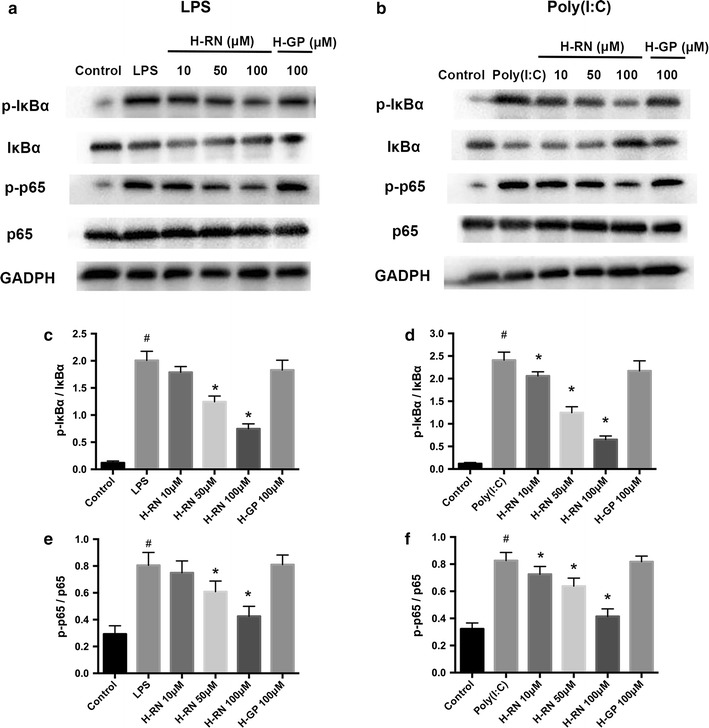
Inhibition of activation of NF-κB signaling pathway by H-RN in corneal fibroblasts induced by LPS or poly(I:C). The effect of H-RN on phosphorylation of IκBα and p65 in corneal fibroblasts induced by LPS (**a**, **c**, **e**) or poly(I:C) (**b**, **d**, **f**) were detected by *western blots.* Data are expressed as mean ± SD. ^#^p < 0.05 compared with control group; *p < 0.05 compared with LPS or poly(I:C) group

NF-κB is a crucial nuclear transcription factor involved in inflammation and immune diseases. To clarify how H-RN affected the NF-κB signaling pathway during corneal inflammation, we detected p65 translocation, phosphorylation of IκBα, and p65 in corneal fibroblasts. The immunofluorescence of reduced translocation of p65 to the nucleus proved that NF-κB activation was inhibited by H-RN either in LPS or poly(I:C) treated corneal fibroblasts (Fig. 6). The protein determination by Western blot found that phosphorylation of IκBα and p65 were both significantly increased by induction of LPS as well as poly(I:C), but were noticeably decreased after the intervention of H-RN (Fig. 7).

### Inhibition of MAPK signaling pathway activated by LPS and poly(I:C)

**Fig. 8:**
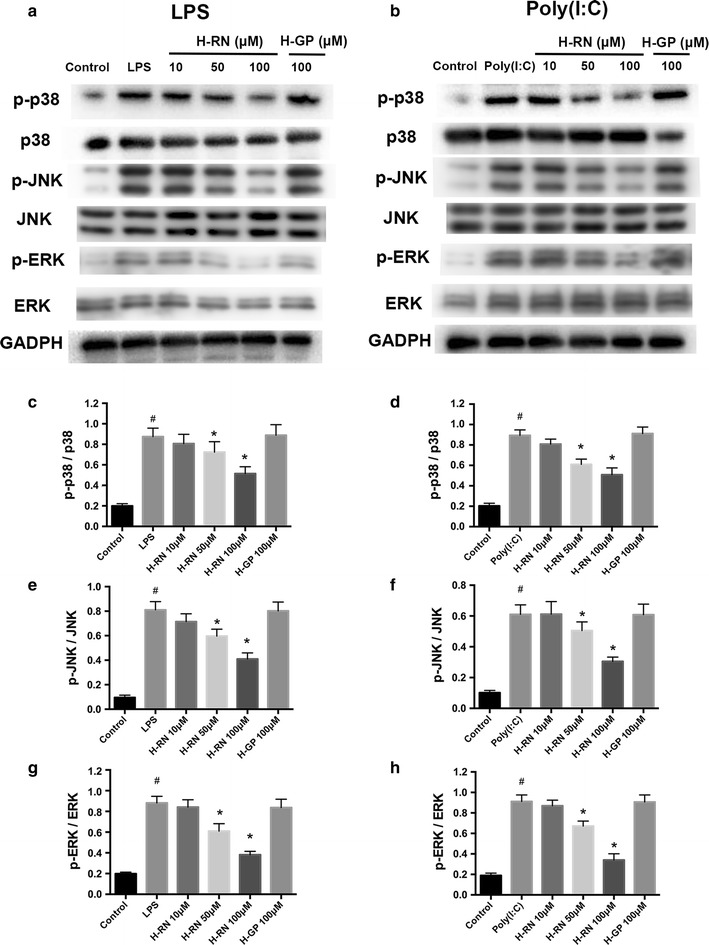
Suppression of MAPK signaling pathway by H-RN in corneal fibroblasts stimulated by LPS or poly(I:C). Expressions of the three participants (p38, JNK, and ERK) of MAPKs in corneal fibroblasts with or without H-RN intervention after stimulation with LPS (**a**, **c**, **e**, **g**) or poly(I:C) (**b**, **d**, **f**, **h**) were accessed by *Western blots*. Data are expressed as mean ± SD. ^#^p < 0.05 compared with control group; *p < 0.05 compared with LPS or poly(I:C) group

Mitogen-activated protein kinases (MAPKs), including p38, JNK, and ERK, contribute to a series of physiological and pathological processes, such as synthesis of inflammatory mediators. In this study, we assessed the expression of the three participants of MAPKs in LPS- or poly(I:C)-induced corneal fibroblasts with or without H-RN intervention. Figure 8 shows a significantly higher expression of phosphorylated p38, JNK, and ERK after incubation with LPS or poly(I:C), which means the activation of MAPKs. However, by treating H-RN, the phosphorylation of all MAPKs were suppressed with statistical significance. This demonstrates that H-RN markedly inhibited the activation of MAPK signaling in corneal fibroblasts not only triggered by LPS but also by poly(I:C).

### Safety of H-RN in corneal fibroblasts and rat eyes

**Fig. 9:**
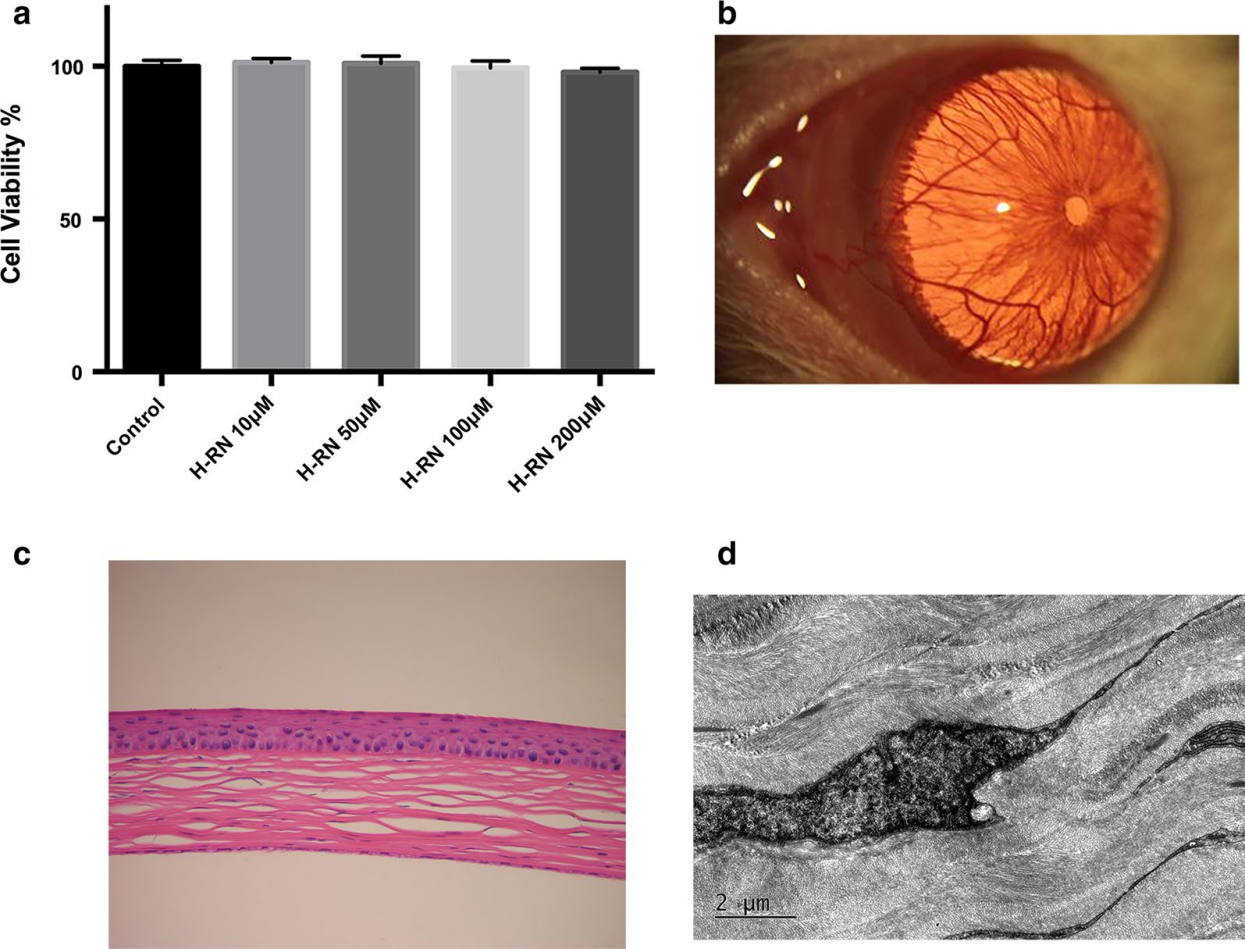
H-RN is non-toxic to corneal fibroblasts or rat eyes. MTS method was used to determine the viability of cells. After incubation with different concentration of H-RN (10, 50, 100, 200 μM,) for 24 h (**a**). In vivo, biomicroscope (**b**), histopathological examination (**c**), and transmission Electron Microscope observation (**d**) were conducted 7 days after rats received subconjunctival injection of 20-μl saline containing 100-μg H-RN

MTS assay showed that incubation with H-RN (10, 50, 100, 200 μM) had no influence on the viability of corneal fibroblasts (Fig. 9a). Moreover, we explored the safety of H-RN by performing biomicroscope, histopathological examination, and Transmission Electron Microscope on the rats and the corneas. No sign of ocular abnormality or cell toxicity was detected in any of the assays (Fig. 9b–d), which demonstrated that H-RN displayed non-toxicity to the cornea.

## Discussion

In keratitis, inflammation often lasts and relapses long after the microbes were eliminated, causing great harm to the structure and function of the cornea. Although bacteria and virus could be killed or limited by antibiotics, the immune system, once activated, won’t stop quickly or immediately and has to be re-regulated by anti-inflammatory drugs due to pathogen products such as LPS and poly(I:C) [17, 18]. This is the most challenging difficulty and troublesome part of the management of keratitis. Among the most commonly used anti-inflammatory drugs, corticoids are preferred, but their profound side-effects (i.e. cataract, high intraocular pressure, difficulty in healing, etc.) have become a major concern [5, 19]. An ideal drug should display both desired efficiency and satisfactory safety.

Theoretically, endogenous human proteins are better tolerated with much less irritation and toxicity to the host, but the large molecular weight and high cost have limited their use in ocular disease. Advantages of small peptides are obvious over proteins as small peptides have lower toxicity and immunology, higher penetration, and lower cost [6, 20]. Also, peptides derived from human endogenous proteins exert potential biofunctions from the original protein as well as unexplored activities due to the sequence and structure. Therefore, we focus on the screening and development of such peptide agents from human endogenous proteins with certain bioactivities to target ocular diseases.

H-RN is a 1254.34-Da peptide (sequence RNPRGEEGGPW), which we identified with biological information technology from human hepatocyte growth factor (HGF), a protein demonstrated to regulate autoimmune and inflammatory diseases [8–10]. Here, in this study, we prove for the first time that H-RN effectively attenuated LPS- and poly(I:C)-induced corneal inflammation in vivo and in vitro and its effects were mediated by suppressing the NF-κB and MAPK/p38/JNK/ERK pathways. The effect is in a dose- and sequence-dependent manner.

Both LPS, surrogate for bacteria, and poly(I:C), an analog of the virus, were used in our study. It is accepted that the two agents are strong stimuli to the immune system and activate Toll-like receptors 3 (TLR3) and TLR4 [21, 22]. TLRs are major pattern receptors that recognize multiple microbial products and mediate inflammatory reactions through producing various chemokines and cytokines to participate in the clearance of the microbes and to defend the host. Meanwhile, the enduring process breaks the homeostasis and destroys the physiological structure essential to the normal function [23, 24]. Among the mediators involved in the TLRs transduction, IL-6, MCP-1, and IFN-γ play crucial roles [3, 25, 26]. When corneal fibroblasts were infected with bacteria or virus and activated by the stimulation, those cytokines were released rapidly as TLRs respond to the microbial components. We quantified the mRNA and protein level of IL-6, MCP-1 and IFN-γ after LPS or poly(I:C) stimulation, and found that peptide H-RN effectively attenuated the increased expressions of those mediators. Furthermore, we demonstrated with different means that the expression of ICAM-1 triggered by LPS or poly(I:C) was significantly inhibited by treatment of H-RN, which, together with the above results, suggested that H-RN could limit the degree of inflammatory reaction by suppressing the principle cytokines, chemokines, and adhesion molecules. One of the main effects that those mediators produce is the recruitment and infiltration of various leukocytes to the cornea. Those infiltrated inflammatory cells, together with activated corneal fibroblasts, release cytotoxic agents and proteases to aggressively clear the pathogens. However, at the same time, the process unavoidably causes extensive damage to the cornea by degrading the stromal collagen and destroying corneal integrity and clarity. It has been implied that depletion of leukocytes or suppression of adhesion molecules resulted in relieved corneal inflammation and less destruction [2]. So, next, we verified H-RN’s in vivo efficiency in LPS-induced keratitis and proved that H-RN effectively attenuated the clinical symptoms in rats and greatly decreased the infiltration of inflammatory cells and abnormalities in the cornea. Also, IL-6, MCP-1, and IFN-γ were also significantly reduced in rat corneas after H-RN injection or topical application. The in vivo results are by and further validated findings of in vitro assays. Moreover, previously we have demonstrated HRN’s effectiveness in inhibiting corneal neovascularization [27]. Since inflammatory disease are often accompanied by angiogenesis, it is highly believed that HRN is protective to corneal inflammation not only due to its anti-inflammation role but also its anti-angiogenic activity.

To illuminate the mechanisms of H-RN’s effect on keratitis, we investigated the possible signaling pathways involved in inflammation. It is recognized that NF-κB and MAPK signals are principle pathways of the cornea to regulate the cytokines and cells to participate in the inflammatory process [25, 28, 29]. Insults such as LPS and poly(I:C) are strong activators to TLRs, which then trigger the NF-κB and MAPK pathways to defense the host. NF-κB is believed to be a crucial initiative factor regulating inflammatory gene expression. It is activated by degradation of IκBα, the inhibitor protein of NF-κB, and then translocates from cytoplasm to the nucleus to promote target genes of the immune system [30]. MAPKs also play an important role in cellular activities including inflammation, innate immunity, and apoptosis. They are a family of serine/threonine protein kinases mainly consisting of JNK, p38, and ERK. It has been demonstrated that activation of TAK1, an upper member of TLR signaling, triggers both the NF-κB and the MAPK pathway [31]. In regards to this, we tested the expression of the main proteins involved in the two pathways to clarify the mechanism underlying H-RN’s anti-inflammatory property in keratitis. As we proved in this study, H-RN effectively inhibited the activation of NF-κB and MAPK signaling by reduction of p65 translocation and suppression of phosphorylation of IκBα, p65, ERK, p38, and JNK, which explains the protective role of H-RN in keratitis. Meanwhile, because the NF-κB and MAPK pathways share some of the top regulators and cross-talks on the TLR-signaling pathway, it is possible that H-RN affected certain such shared signals, which needs to be studied further and is under active investigation in our next work.

The safety of the drug in ocular tissues is a major consideration. We conducted safety evaluations with biomicroscopes, histological observations, and transmission electron microscopes after subconjunctival injection of H-RN, as well as cell viability measures on corneal fibroblasts. No sign of clinical irritation or damage in the ultrastructure was shown. Also, no difference in cell viability was noticed in corneal fibroblasts incubated with H-RN. From what we observed in the study, H-RN is non-toxic to the eye, which is consistent with the fact that it is an endogenous derivation. Certainly, more examinations remain to be finished in the future to comprehensively verify H-RN’s effects other than its anti-inflammatory property.

Since H-RN is derived from endogenous protein and has only 11 amino acids, it is much more cost-effective and has better penetration ability than large proteins, as well as lower irritation than chemical drugs. But the half-life may be short with such a small peptide and the penetration of H-RN in different ocular tissues still needs to be proved, which are limitations of the present study. Also, there is no mature animal model other than LPS-induced keratitis available to further explore the effect of H-RN on corneal inflammation. Virus-stimulated animal keratitis does not fit in our study as H-RN is not an anti-virus agent and it mainly targets the immune response during inflammation rather than killing the microbes. For this reason, the in vivo model of LPS-induced keratitis was chosen in our study. It should be noted that subconjunctival injection showed better efficiency than topical drops of H-RN, indicating that a longer-acting time may increase the benefits of this small peptide. Although the present data did not show a better effect of H-RN as compared with DEX, the potential of H-RN will be further developed as it is now only a pure peptide and not yet modified. It is suggested that with a proper modification method and an improved delivery system, the efficiency of the peptide drug will be strengthened [32, 33].

## Conclusion

In conclusion, we have shown that the novel peptide H-RN, which we derived from HGF, effectively attenuated keratitis in vivo and in vitro through blocking the NF-κB and MAPK signaling pathways. Therefore, H-RN exhibits great potential as a promising and safe agent in the future in treating keratitis.

## Abbreviations

HGF: hepatocyte growth factor
ICAM-1: intercellular cell adhesion molecule-1
MCP-1: monocyte chemotactic protein-1
DEX: dexamethasone
H&E: hematoxylin and eosin
MTS: 3-(4,5-dimethylthiazol-2-yl)-5-(3-carboxymethoxypehenol)-2-(4-sul-phophenyl)-2H-tetrazolium
